# Social exclusion, thwarted belongingness, and perceived burdensomeness: construct validity and psychometric properties of the Interpersonal Needs Questionnaire among patients with sexually transmitted infections in Shanghai, China

**DOI:** 10.1186/s40359-022-00726-7

**Published:** 2022-02-14

**Authors:** Ruijie Gong, Suping Wang, Yucheng Ji, Zhile Li, Ruijie Chang, Shuxian Zhang, Xiaoyue Yu, Chen Xu, Yong Cai, Yang Ni

**Affiliations:** 1 Shanghai Xuhui Center for Disease Control and Prevention, No. 50 Yongchuan Road, Shanghai, 200237 China; 2 School of Public Health, Shanghai Jiao Tong University School of Medicine, No. 227 South Chongqing Road, Shanghai, 200025 China; 3 School of Medicine, Shanghai Jiao Tong University, No. 227 South Chongqing Road, Shanghai, 200025 China; 4 Shanghai Skin Disease Hospital, No. 1278 Baode Road, Shanghai, 200443 China

**Keywords:** Social exclusion, Thwarted belongingness, Perceived burdensomeness, Interpersonal need, Interpersonal Needs Questionnaire, Sexually transmitted infections

## Abstract

**Background:**

Sexually transmitted infections (STIs) are a serious public health problem worldwide. Patients with STIs have a high rate of psychosocial problems and may perceive unmet interpersonal needs, which is considered a proximal and sufficient cause of suicidal thoughts and behaviors. The present study examined the construct validity and psychometric properties of the 15-item Interpersonal Needs Questionnaire among patients with STIs in Shanghai, China.

**Methods:**

We recruited 910 patients with STIs (438 males and 472 females; mean age = 38.72, standard deviation [SD] = 13.034) from the Shanghai Skin Disease Hospital using accidental sampling. Baseline descriptive statistics were calculated using R 4.0.0, and a latent variable model was developed using Mplus 7.4.

**Results:**

The construct validity results supported a latent variable measurement model with three distinct but related constructs (thwarted belongingness, perceived burdensomeness, and social exclusion) (p < 0.001, χ^2^/df = 2.475, root mean square error of approximation = 0.057, comparative fit index = 0.931, Tucker–Lewis index = 0.916, standardized root mean residual = 0.044). The Cronbach’s α and McDonald’s ω values were 0.849 and 0.767 for the total scale, 0.888 and 0.889 for perceived burdensomeness, 0.764 and 0.777 for social exclusion, and 0.892 and 0.893 for thwarted belongingness. Interpersonal needs were significantly associated with low self-esteem (r = 0.539), loneliness (r = 0.573), depression (r = 0.338), entrapment (r = 0.420), defeat (r = 0.579), and low perceived social support (r = 0.424).

**Conclusions:**

This was the first study to highlight social exclusion as a distinct but related dimension of interpersonal needs. This finding indicates that patients with STIs perceive high social exclusion. Therefore, health providers should consider the psychological status of these patients and implement strategies to support their integration into society.

**Supplementary Information:**

The online version contains supplementary material available at 10.1186/s40359-022-00726-7.

## Background

**Fig. 1:**
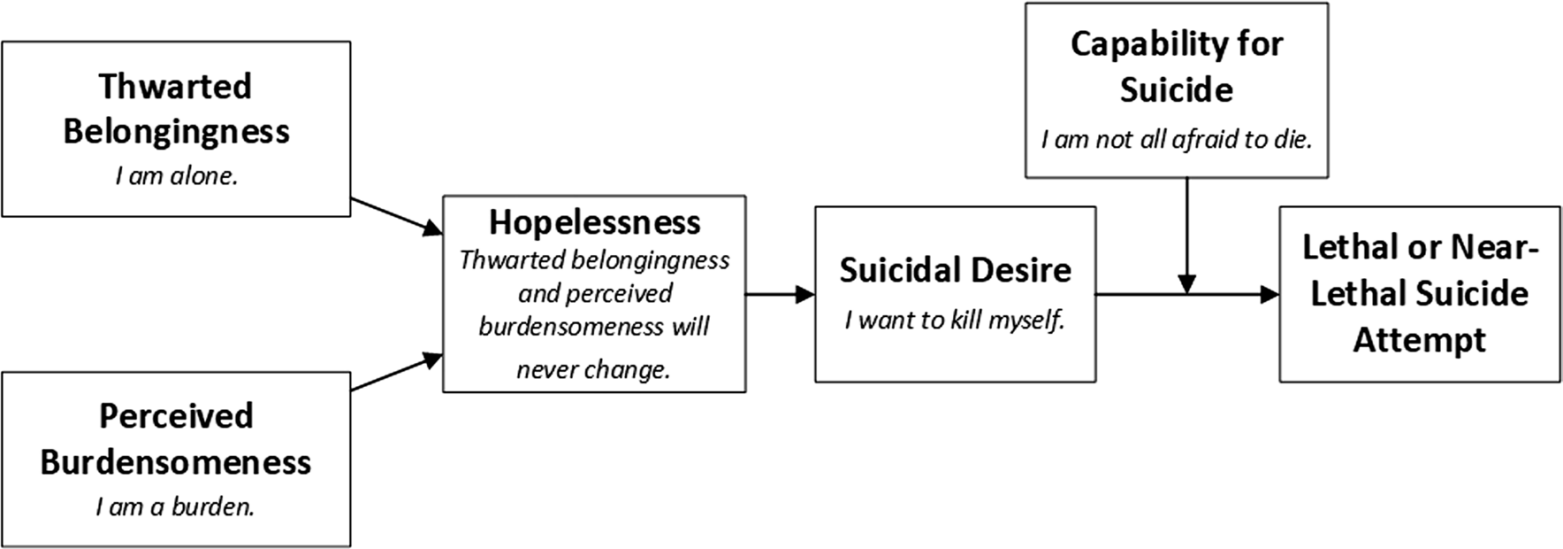
Causal pathways from the perspective of the Interpersonal Theory of Suicide

Sexually transmitted infections (STIs) are a serious public health problem worldwide. This problem is associated with an economic burden on society as well as physical harm and psychosocial burdens for affected patients [1]. Most available STI-related research has focused on prevalence [2, 3], epidemiology [4, 5], health services [6, 7], and prevention [1, 8]. However, many studies have demonstrated high rates of psychosocial problems among patients with STIs [9, 10], including low self-esteem, low self-efficacy, denying personal values [3], stigma, shame [11], depression [12–14], and anxiety [12]. Loneliness, lack of reciprocal care, and unmet needs for social competence may result in perceived burdensomeness [15] and an unmet need to belong. This social disconnection may lead to thwarted belongingness [16, 17], which also comprises an unmet interpersonal need [18, 19]. In turn, unmet interpersonal needs can exacerbate low self-esteem, loneliness, lack of social support [20], and depression [21]. Cramer [22] recently showed that unmet interpersonal needs were associated with defeat (e.g., failing to achieve important goals and experiencing a loss in social rank) [23] and entrapment (e.g., lack of available options for escape from an aversive situation) [23], which were the two main constructs of the Integrated Motivational-Volitional (IMV) Model of suicide [24]. Meanwhile, the interpersonal theory of suicide [18] suggests that thwarted belongingness and perceived burdensomeness are proximal and sufficient causes of suicidal thoughts and behaviors (Fig. 1). Therefore, it is important to measure interpersonal needs among patients with STIs, especially as around 25% of these patients are reported to have suicidal thoughts [9].

The Interpersonal Needs Questionnaire (INQ) has been used to assess interpersonal needs [21] among adolescents [25], college students [26–28], adults [29], older adults [26], and patients receiving psychiatric care [30–32]. The INQ items were drawn from Baumeister and Leary’s definition of the need to belong [17], inclusionary status as proposed by Leary [33], and the Mattering to Others Questionnaire developed by Marshall [34]. Although a two-factor structure (thwarted belongingness and perceived burdensomeness) for the INQ showed a reliable and acceptable fit with college students, some items adapted from the inclusionary status model overlap with content regarding social exclusion and extend beyond the concept of belongingness [20]. The term “social exclusion” is closely associated with “social inclusion,” and is described as the state of disadvantage faced by particular groups who are perceived as removed from mainstream society and who cannot fully participate in normal life [35]. STIs are often associated immoral or irresponsible behavior in the context of sociocultural norms [36]. Therefore, it is logical that social exclusion can encompass patients with STIs who had experienced being stigmatized and marginalized [37]. This means it is important to assess whether the two-factor structure of the INQ is appropriate for patients with STIs.

This study represents the first time the INQ has been used in patients with STIs. The purpose of this study was threefold: 1) to examine the construct validity and reliability of the INQ in this population; and to assess the convergent and divergent validity of the INQ in this population.

## Methods

### Study procedure

This study was approved by the Shanghai Jiao Tong University School of Medicine Public Health and Nursing Ethics Committee. The procedure for the present study has been detailed in a previous report [9]. This study used a cross-sectional design and was conducted from November 2017 to December 2018. Written informed consent was obtained from all participants before the investigation started.

### Participants

**Fig. 2:**
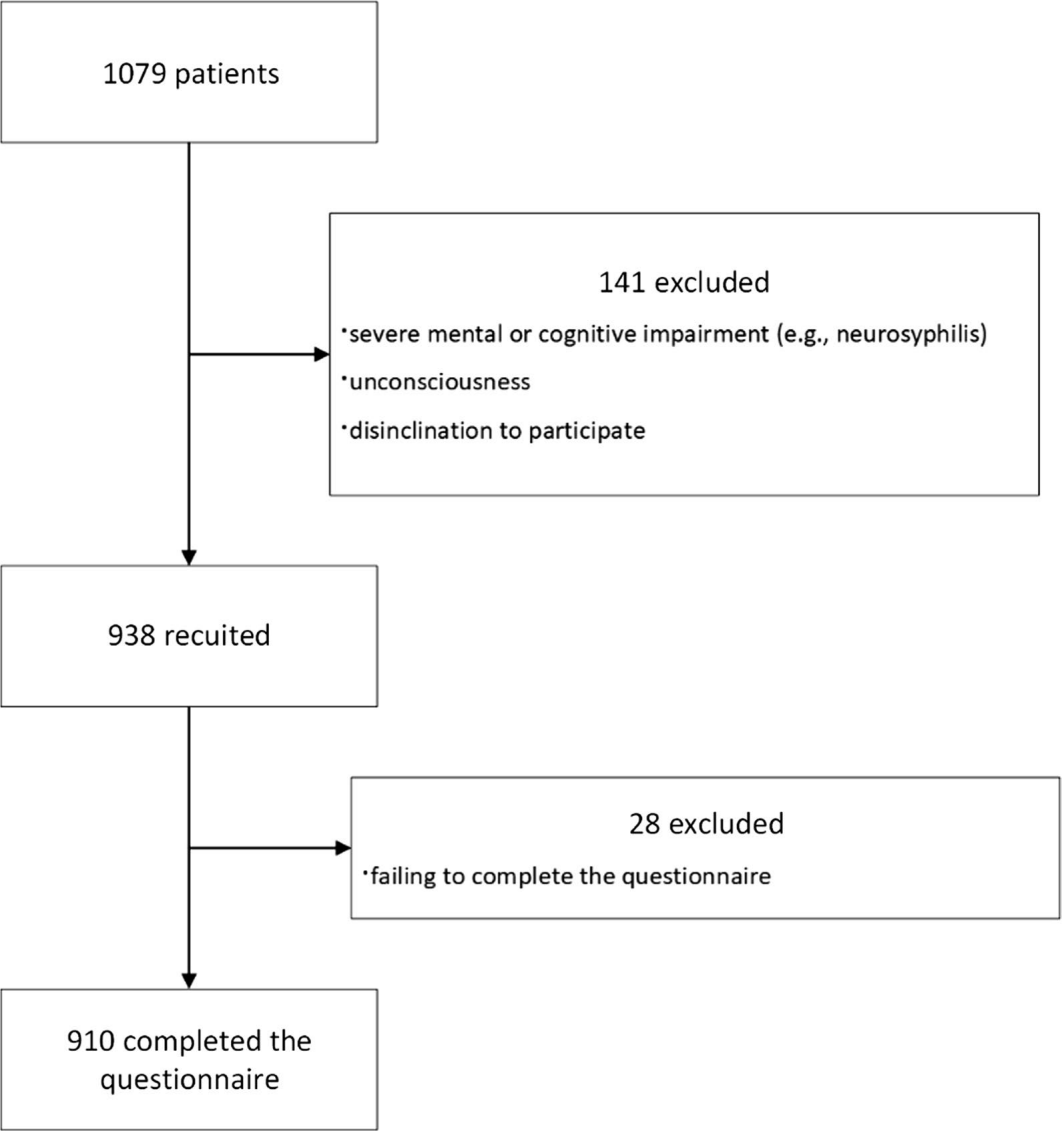
Flow of participants

Potential participants were patients who visited one of the two branch institutes of the Shanghai Skin Disease Hospital (Qiujiang Road Branch and Baode Road Branch) to consult a doctor for STI treatment on a Wednesday or Saturday (when our investigators were present). The inclusion criteria were: age ≥ 18 years, clinical diagnosis of an STI, able to read and sign the informed consent form, and no participation in a similar study in the previous 6 months. The exclusion criteria were: severe mental or cognitive impairment (e.g., neurosyphilis), unconsciousness, or disinclination to participate. Figure 2 shows the flow of participants in this study.

In total, we included 910 patients using accidental (convenience) sampling [9]; 438 were male and 472 were female. Participants’ ages ranged from 18 to 83 years, with a mean age of 38.72 years (SD = 13.034). The 910 participants were randomly split in two groups with an equal number of participants. The first group (Sample 1) was used for the exploratory factor analysis (EFA), and the second group (Sample 2) was used for confirmatory factor analysis (CFA). In Sample 1, 231 participants were male and 224 were female, and the mean age was 38.58 years (SD = 13.298). In Sample 2, 207 participants were male and 248 were female, and the average age was 38.85 years (SD = 12.777).

### Measures

#### Chinese version of the INQ

The original English-language version of the INQ had 25 items. The INQ has been translated into several other languages and published, including Chinese [21, 26, 27]. A 15-item version of the INQ (INQ-15), which was published by the original authors and translated by Chen, has been validated in both college students and older adults [26]. In the INQ-15, each item is assessed on a 7-point Likert scale. Six items (items 7, 8, 10, 13, and 14) are reverse scored. The scale consists of two subscales—perceived burdensomeness (including Item 1 to Item 6) and thwarted belongingness (including Item 7 to Item 15). A higher score indicates greater severity of unmet interpersonal needs (Cronbach’s α = 0.849).

#### Chinese version of the Multidimensional Scale of Perceived Social Support (MSPSS)

The MSPSS is a 12-item self-administered measure of perceived social support, with responses on a 7-point Likert-type scale (from 1 = strongly disagree to 7 = strongly agree) [38]. The scale distinguishes perceived social support from three sources (family, friends, and significant others), and is an extensively translated and validated social support outcome measure [39]. The Chinese version of the MSPSS has good validity and reliability [40]. The “significant others” dimension is measured by items 1, 2, 5, and 10 (Cronbach’s α = 0.914). The “family” dimension is measured by items 3, 4, 8, and 11 (Cronbach’s α = 0.889). Finally, the “friends” dimension is measured by items 6, 7, 9, and 12 (Cronbach’s α = 0.921). A higher total score for all items indicates higher perceived social support (Cronbach’s α = 0.947).

#### Chinese version of the 8-item UCLA Loneliness Scale (ULS-8)

The ULS-8 is a self-report measure of perceived loneliness. Hays refined an 8-item version of the scale to achieve similar reliability to the original 20-item scale while reducing the time burden on respondents [41]. Respondents are asked to report the frequency with which they experience dissatisfaction and satisfaction with social relationships from 1 (“never”) to 4 (“always”), with two items (items 3 and 6) being reverse scored. The Chinese version of the ULS-8 was translated and published by Zhou and showed good validity and reliability in both older adults and adolescents [42, 43]. A higher score suggests higher perceived loneliness (Cronbach’s α = 0.813).

#### Chinese version of the 9-item Patient Health Questionnaire (PHQ-9)

The PHQ-9 is an instrument for making diagnoses and assessing the severity of depressive disorders in the past 2 weeks, which uses the nine diagnostic criteria for depressive disorders in the Diagnostic and Statistical Manual of Mental Disorders, Fourth Edition [44, 45]. The Chinese version of the PHQ-9 has satisfactory validity and reliability [46]. A higher score represents a higher degree of depressive symptoms (Cronbach’s α = 0.909).

#### Chinese version of the Rosenberg Self-esteem Scale (RSES)

The 10-item RSES is rated on a 4-point Likert type scale from 1 (“completely disagree”) to 4 (“completely agree”), and is used to assess the degree of self-esteem [47]. Five items (items 3, 5, 8, 9, and 10) are reverse scored. The Chinese version of the RSES has been widely used [48]. The first five items measure self-competence (Cronbach’s α = 0.761) and the remaining five items measure self-liking (Cronbach’s α = 0.721) [26]. A higher score indicates a higher level of self-esteem (Cronbach’s α = 0.763).

#### Chinese version of the Entrapment Scale (ES)

The ES is a 16-item measure that reflects the escape motivation triggered by either perceptions of the outside world or inner feelings in the past week [23]. Responses are on a 5-point Likert-type scale from 0 (“not at all like me”) to 4 (“extremely like me”). Items 1–10 cover external entrapment (Cronbach’s α = 0.944), and items 11–16 cover internal entrapment (Cronbach’s α = 0.939). The Chinese version of the ES has satisfactory validity and reliability in college students [49]. Higher scores indicate greater feelings of entrapment (Cronbach’s α = 0.966).

#### Chinese version of the Defeat Scale (DS)

The DS is a 16-item measure of perceptions of failed struggles and low social rank in the past week [23]. Responses are on a 5-point Likert-type scale from 0 (“never”) to 4 (“always”). Three items (items 2, 4, and 9) are reverse scored. The Chinese version of the DS has been translated and used with college students [50]. Higher scores indicate greater feelings of defeat (Cronbach’s α = 0.920).

### Statistical analyses

Statistical analyses were performed using Mplus version 7.4 for Windows (Muthen & Muthen, USA) and R 4.0.0 for Windows (R Core Team, Austria). Baseline descriptive statistics were used to summarize participants’ demographic characteristics, INQ items, and psychosocial factors using R 4.0.0. EFA was conducted using Mplus Version 7.4. Although the 15 items were continuous variables, the majority of these items were not normally distributed. Maximum likelihood estimation with robust standard errors (MLR) with geomin oblique rotation was used for transformations as necessary. The fit indices used were: chi-square (χ^2^), standardized root mean square residual (SRMR), comparative fit index (CFI), Tucker–Lewis index (TLI), and the root mean squared error of approximation (RMSEA). MLR was used for the CFA.

A structural equation model with six observed variables regressed onto the INQ-15 measurement model was run using MLR. All observed variables were regressed onto latent variables to examine the magnitude and direction of the regression coefficients. In addition, all observed variables in the structural part of the model were covariates.

## Results

### Description of the INQ-15

Descriptive statistics (i.e., number, mean, standard error [SE], SD, variance, skew, kurtosis, and range) for the 15 items were calculated for the total sample, as well as the inter-correlations among these variables (Additional file 1: Table S1).

### Construct validity

#### EFA

**Fig. 3:**
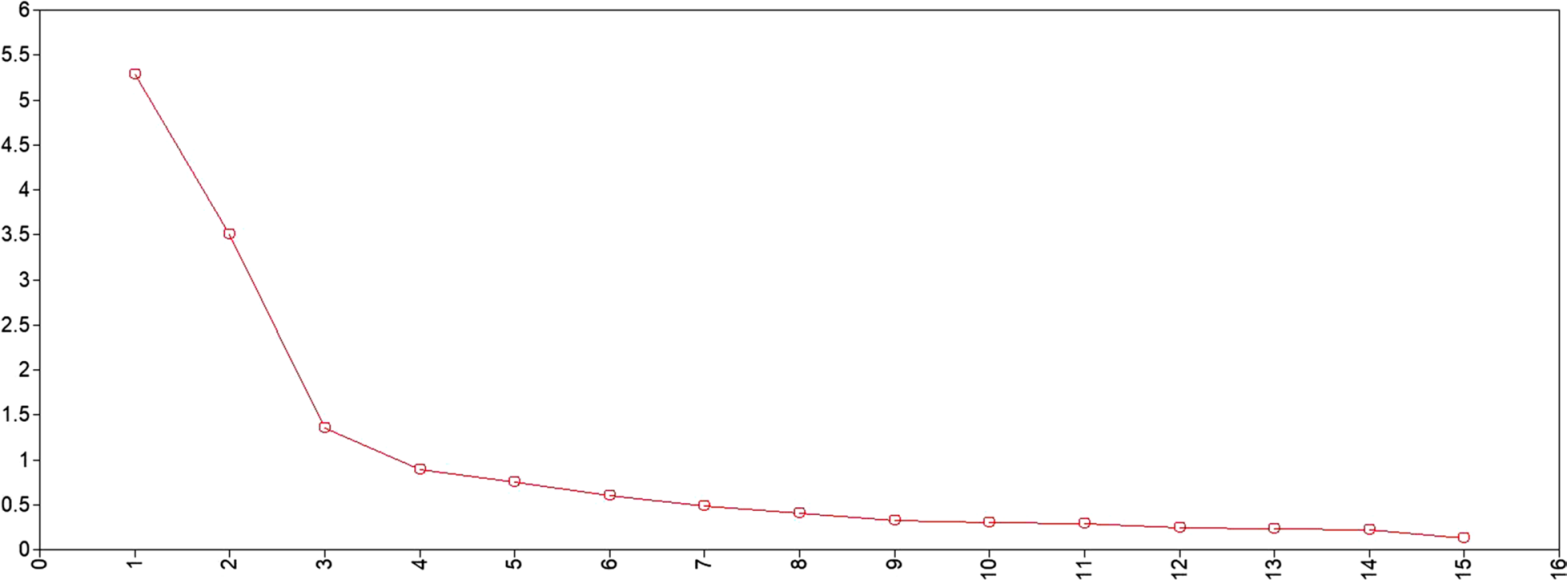
Eigenvalues from factor analysis

The eigenvalues for Sample 1 (Table 1) showed three eigenvalues greater than 2, and a four-factor model provided a good fit to the data. However, the factor loading showed that a three-factor model had good indicators for each factor (Table 2 and Fig. 3). The pattern of loadings for this model indicated that the six items that measured perceived burdensomeness loaded on the “burdensomeness” factor, and six of the nine thwarted belongingness items loaded onto the “belongingness” factor. The remaining three items (“These days, I rarely interact with people who care about me”; “These days, I feel disconnected from other people”; and “These days, I often feel like an outsider in social gatherings”) loaded on both the “social exclusion” and “belongingness” factors. These items did not fit the criteria proposed by Muthén and Muthén that an item should show a factor loading two times greater on the relevant factor than the loading on another factor (as well as being statistically significant) [51]. Therefore, the three-factor model was considered.

**Table 1:** Fit statistics for exploratory and confirmatory factor analysis models

Model	Factor	x^2^	df	x^2^/df	p	RMSEA	90% CI	CFI	TLI	SRMR	Eigenvalue
EFA for sample1	1	1375.780	90.000	15.286	< 0.001	0.177	0.169–0.186	0.405	0.306	0.195	5.288
	2	598.996	76.000	7.882	< 0.001	0.123	0.114–0.132	0.758	0.666	0.070	3.508
	3	463.786	63.000	7.362	< 0.001	0.118	0.108–0.128	0.815	0.691	0.046	1.351
	4	193.466	51.000	3.793	< 0.001	0.078	0.067–0.090	0.934	0.864	0.029	0.896
CFA for sample2	3	403.177	87.000	4.634	< 0.001	0.089	0.081–0.098	0.829	0.793	0.067	
	3-Refined	212.884	86.000	2.475	< 0.001	0.057	0.047–0.067	0.931	0.916	0.044

**Table 2:** Standardized factors for a three-factor model in sample 1 (exploratory factor analysis)

Item	Perceived burdensomeness	Thwarted belongingness	Social exclusion
*These days, ······*			
1 The people in my life would be better off if I were gone	0.721*	− 0.002	− 0.047
2 The people in my life would be happier without me	0.711*	− 0.031	− 0.048
3 I think I am a burden on society	0.815*	0.023	0.010
4 I think my death would be a relief to the people in my life	0.868*	− 0.014	− 0.047
5 I think the people in my life wish they could be rid of me	0.827*	0.002	0.028
6 I think I make things worse for the people in my life	0.713*	0.023	0.184*
7 Other people care about me	0.039	0.712*	0.061
8 I feel like I belong	− 0.015	0.720*	− 0.013
9 These days, I rarely interact with people who care about me	0.446*	0.046	0.537*
10 These days, I am fortunate to have many caring and supportive friends	0.010	0.785*	− 0.060
11 These days, I feel disconnected from other people	0.536	− 0.025	0.537*
12 These days, I often feel like an outsider in social gatherings	0.462*	− 0.006	0.687*
13 These days, I feel that there are people I can turn to in times of need	− 0.022	0.847*	0.292*
14 These days, I am close to other people	0.029	0.829*	0.001
15 These days, I have at least one satisfying interaction every day	0.025	0.691*	− 0.061

*p<0.05

#### CFA

Fit statistics for the CFA are presented in Table 1. The fit statistics for the three-factor model in Sample 2 were not at acceptable levels; therefore, that model was refined according to the modification index. The SRMR and RMSEA values for the refined model indicated an adequate-to-good fit (i.e., < 0.08), and the CFI and TLI values showed an acceptable fit (i.e., 0.90). Although the chi-square test of model fit was inconsistent with an adequate fit (i.e., χ^2^/df < 2.00), this index is overly sensitive to sample size; given the large samples used in this study (n = 455); therefore, the chi-square test might have underestimated the degree of model fit [52].

**Fig. 4:**
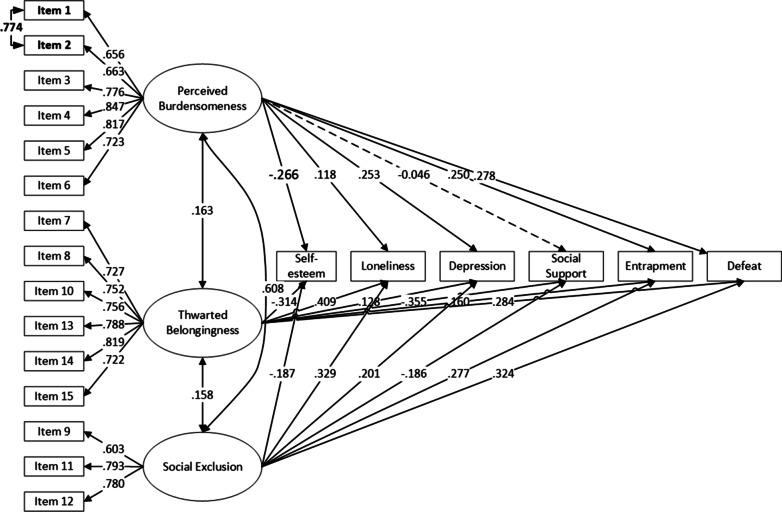
Structural equation model results examining discriminant relationships. Loneliness = 8-item UCLA Loneliness Scale; Defeat = Defeat Scale; Entrapment = Entrapment Scale; Depression = 9-item Patient Health Questionnaire; Social Support = Multidimensional Scale of Perceived Social Support; Self-esteem = Rosenberg Self-esteem Scale. Dotted line indicates statistically insignificant loadings, all other loadings were significant at p < .05

Parameter estimates for the refined model (standardized estimated factor loadings, SE, covariances, and R^2^ values [i.e., communalities]) are presented in Table 3 and Fig. 4. All items significantly loaded onto the specified latent variable, with r^2^ values from 0.25 to 0.69; most values fell in the middle, indicating moderate magnitudes (i.e., 0.30–0.60). The three factors (perceived burdensomeness, thwarted belongingness, and social exclusion) were distinct but related, with social exclusion and perceived burdensomeness being closely related.

**Table 3:** Model estimated factor loadings, covariances, and P-values for Sample 2

	Estimated	SE	p	R-sq
*Perceived burdensomeness*				
Item1	0.662	0.063	< 0.001	0.438
Item2	0.695	0.063	< 0.001	0.483
Item3	0.684	0.067	< 0.001	0.468
Item4	0.829	0.036	< 0.001	0.688
Item5	0.782	0.048	< 0.001	0.611
Item6	0.689	0.053	< 0.001	0.474
*Thwarted belongingness*				
Item7	0.754	0.037	< 0.001	0.568
Item8	0.773	0.033	< 0.001	0.598
Item10	0.747	0.033	< 0.001	0.558
Item13	0.794	0.026	< 0.001	0.630
Item14	0.817	0.027	< 0.001	0.668
Item15	0.736	0.032	< 0.001	0.542
*Social exclusion*				
Item9	0.504	0.058	< 0.001	0.254
Item11	0.753	0.054	< 0.001	0.566
Item12	0.801	0.045	< 0.001	0.641
*Covariances*				
Item1 WITH Item2	0.773	0.059	< 0.001	
SE WITH PB	0.544	0.065	< 0.001	
SE WITH TB	− 0.105	0.052	< 0.001	
PB WITH TB	− 0.172	0.048	< 0.001

### Reliability

The Cronbach’s α and McDonald’s ω values for the Chinese version of the INQ-15 were 0.849 and 0.767 for the total scale, 0.888 and 0.889 for perceived burdensomeness, 0.764 and 0.777 for social exclusion, and 0.892 and 0.893 for thwarted belongingness.

### Psychometric properties and convergent and divergent validity

#### Description of psychosocial factors

Descriptive statistics (i.e., number, mean, SE, SD, variance, skew, kurtosis, and range) were calculated for the psychosocial factors (i.e., perceived social support, loneliness, depression, self-esteem, entrapment, and defeat) for the total sample (Table 4). Self-esteem, depression, entrapment and defeat were weakly associated with perceived burdensomeness, thwarted belongingness and social exclusion. Loneliness was weakly associated with perceived burdensomeness and social exclusion, while median associated with thwarted belongingness. Social support was weakly associated with thwarted belongingness and social exclusion.

**Table 4:** Descriptive statistics for psychosocial factors and relationships with interpersonal needs

Psychosocial factors	Mean	Median	SD	Range	Skew	Kurtosis	SE	Interpersonal need		
								TB	PB	SE
Interpersonal need	36.33	35.00	14.63	81.00	0.40	− 0.43	0.49	0.838**	0.653**	0.595**
TB	19.96	19.00	9.92	36.00	0.47	− 0.58	0.33	1.000	0.272**	0.238**
PB	9.75	6.00	6.35	36.00	2.24	5.54	0.21	0.272**	1.000	0.546**
SE	6.63	5.00	4.23	18.00	1.16	0.68	0.14	0.238**	0.546**	1.000
Perceived Social support	63.48	69.00	14.67	72.00	− 1.03	1.22	0.49	− 0.410**	− 0.269**	− 0.260**
Significant others	21.62	24.00	5.69	24.00	− 1.12	0.84	0.19	− 0.373**	− 0.213**	− 0.216**
Family	22.45	24.00	4.96	24.00	− 1.21	1.82	0.16	− 0.378**	− 0.254**	− 0.249**
Friends	21.41	23.00	5.67	24.00	− 1.02	0.74	0.19	− 0.393**	− 0.235**	− 0.234**
Loneliness	14.72	14.00	4.88	24.00	0.53	− 0.22	0.16	0.498**	0.403**	0.435**
Depression	6.19	5.00	5.39	27.00	1.38	2.50	0.18	0.236**	0.369**	0.324**
Self-esteem	29.80	29.00	4.64	28.00	0.27	0.18	0.15	− 0.412**	− 0.435**	− 0.369**
Self-competence	15.05	15.00	2.56	15.00	− 0.02	0.49	0.08	− 0.387**	− 0.399**	− 0.346**
Self-liking	14.57	15.00	2.52	15.00	0.16	0.23	0.08	− 0.359**	− 0.389**	− 0.310**
Entrapment	12.12	7.50	13.30	64.00	1.18	1.02	0.44	0.285**	0.441**	0.404**
External	7.75	5.00	8.36	40.00	1.16	1.01	0.28	0.294**	0.426**	0.395**
Internal	4.37	2.00	5.33	24.00	1.26	1.02	0.18	0.242**	0.438**	0.387**
Defeat	16.50	14.00	11.15	60.00	0.89	0.42	0.37	0.434**	0.509**	0.463**

TB = Thwarted belongingness, PB = Perceived burdensomeness, SE = Social exclusion

**p < 0.01

#### Convergent and divergent validity

Based on previous findings, we posited that self-esteem, loneliness, and social support were related to thwarted belongingness; self-esteem and depression were related to perceived burdensomeness; and loneliness, entrapment, and defeat were related to social exclusion.

Figure 4 shows the standardized regression coefficients for the simultaneous regression of the six psychosocial factors on the latent variables of perceived burdensomeness, thwarted belongingness, and social exclusion. The thwarted belongingness and social exclusion dimensions were associated with self-esteem(r = − 0.314, − 1.87), loneliness(r = 0.409, 0.329), depression(r = 0.128, 0.201), social support(r = − 0.355, − 0.186), entrapment(r = 0.160, 0.277), and defeat (r = 0.284, 0.324). In addition, perceived burdensomeness was significantly associated with self-esteem (r = -0.266), loneliness (r = 0.118), depression (r = 0.253), entrapment (r = 0.250), and defeat (r = 0.278), but not with social support.

## Discussion

Our results for the construct validity of the Chinese version of the INQ-15 supported the viability of a latent variable measurement model of the INQ-15 for patients with STIs. This model had six indicators for the perceived burdensomeness dimension, six indicators for the thwarted belongingness dimension, and three indicators for the social exclusion dimension. As the INQ-15 was developed as an English language instrument for measuring interpersonal needs, cultural adaptation of the scale should be considered. To our knowledge, only two studies have validated the Chinese version of the INQ-15 in college students and older adults [26, 53]; both studies showed only two dimensions of interpersonal needs (perceived burdensomeness and thwarted belongingness), which was consistent with findings in other countries (i.e., the US [20], Korea [54], Australia, Germany [55], Italy [56], and Slovenia [57]). Therefore, language and culture should not be confounding factors. Previous empirical studies among college students [26–28], older adults [26], clinical patients [30–32], and military service members [58] demonstrated that the INQ-15 was a commonly used measure for the structure of thwarted belongingness and perceived burdensomeness, and the scale demonstrated strong convergent validity. However, patients with STIs may differ from previously investigated populations, which were encouraged to develop close interpersonal bonds, especially as context or environment may influence social connections [59]. Therefore, those populations might have perceived high social connections. This means that the dimension of social exclusion was important for patients with STIs. It is worth noting that the items measuring social exclusion (e.g., “These days, I rarely interact with people who care about me”) were only slightly modified from those used to assess inclusionary status [20], and it is possible that social exclusion may exist if a specific population perceives that they are removed from society, irrespective of whether this was based on their free will. When the original 25-item INQ was refined to 15 items, the authors found that in undergraduates, several items (e.g., “These days, I feel disconnected from other people” and “These days, I often feel like an outsider in social gatherings”) shared content related to social exclusion beyond shared content of belongingness [20]. This finding suggested that social exclusion, which can be considered an indicator of a lack of social connections with others, should also be considered as a dimension of interpersonal needs.

The second aim of the present study was to clarify the psychometric properties of the Chinese version of the INQ-15. Consistent with previous research, we demonstrated that thwarted belongingness and perceived burdensomeness were distinct but related dimensions of interpersonal needs [20], and interpersonal needs were correlated with self-esteem, loneliness, social support [20, 54], depression [21, 55], defeat, and entrapment [22]. This study also found evidence for the convergent validity of thwarted belongingness, perceived burdensomeness, and social exclusion. However, the discriminant validity was inadequate for all three dimensions. To our knowledge, two previous studies [20, 26] reported discriminant validity for perceived burdensomeness, but more elaborate analysis was considered necessary to distinguish the two constructs [54]. These findings indicated that further research is needed to clarify the discriminant validity for the INQ-15.

There were several limitations in the present study. First, as the participants were clinical patients with STIs, no follow-up was conducted to evaluate feasibility and sensitivity. Therefore, predictive validity analyses were not performed. Second, this was the first time that social exclusion has been highlighted as a distinct but related dimension of interpersonal needs, in addition to thwarted belongingness and perceived burdensomeness. This may be explained by the high level of social exclusion perceived by patients with STIs. However, further empirical studies in specific groups should be conducted, including among patients with STIs. In addition, the measurements used in this study were self-reported. Objective cognitive tasks should be considered in further studies to extend our findings.

## Conclusion

The present findings indicate that social exclusion, thwarted belongingness, and perceived burdensomeness are distinct but related dimensions of interpersonal needs among patients with STIs. These novel findings provide inspiration for further research and may stimulate discussion on this issue. First, patients with STIs suffer from psychosocial problems as well as physiological problems, especially high levels of perceived social exclusion. Therefore, strategies should be implemented to reduce discrimination against these patients and support their integration into society through improving perceptions of support from family, friends, and significant others. This may encourage them to actively seek treatment, comply with treatment, and stay optimistic. Second, perceived interpersonal needs are closely associated with psychological status. The INQ-15, along with other scales (e.g., the PHQ-9 and USL-8), can be used to evaluate psychological status as part of suicide risk assessment for patients with STIs throughout the entire treatment period. That means that in the process of diagnosis and treatment of patients with STIs, health providers need not only to provide professional medical help, but also pay attention to humanistic care, and assist in diagnosis and treatment with psychiatry when necessary.

## Supplementary Information

**Additional file 1**. Descriptive Statistics and Inter-correlations among the Interpersonal Needs Questionnaire Items.

## Data Availability

The datasets used and/or analysed during the current study are available from the corresponding author on reasonable request.

## Abbreviations

STIs: Sexual transmitted infections
IMV: The Integrated Motivational-Volitional Model of suicide
INQ: Interpersonal Needs Questionnaire
INQ-15: 15-Item version of the Interpersonal Needs Questionnaire
EFA: Exploratory factor analysis
CFA: Confirmatory factor analysis
MSPSS: Multidimensional Scale of Perceived Social Support
USL-8: 8-Item UCLA Loneliness Scale
PHQ-9: 9-Item Patient Health Questionnaire
RSES: Rosenberg Self-esteem Scale
ES: Entrapment Scale
DS: Defeat Scale
χ^2^: Chi-square
MLR: Maximum likelihood estimation with Robust standard errors
SRMR: Standardized root mean square residual
CFI: Comparative Fit Index
TLI: Tucker–Lewis Index
RMSEA: Root mean squared error of approximation
SE: Standard error
SD: Standard deviation
