# Supplementary material for: Social exclusion, thwarted belongingness, and perceived burdensomeness: construct validity and psychometric properties of the Interpersonal Needs Questionnaire among patients with sexually transmitted infections in Shanghai, China
Source: BMC Psychol. 2022 Feb 14;10:29. doi: 10.1186/s40359-022-00726-7 (PMC8842558; doi:10.1186/s40359-022-00726-7)
Supplement: Supplementary file 1 — Additional file 1. Descriptive Statistics and Inter-correlations among the Interpersonal Needs Questionnaire Items. [file 40359_2022_726_MOESM1_ESM.docx]

**Supplement Table 1. Descriptive Statistics and Inter-correlations among the Interpersonal Needs Questionnaire Items**

|  | **Item1** | **Item2** | **Item3** | **Item4** | **Item5** | **Item6** | **Item7** | **Item8** | **Item9** | **Item10** | **Item11** | **Item12** | **Item13** | **Item14** | **Item15** |
| --- | --- | --- | --- | --- | --- | --- | --- | --- | --- | --- | --- | --- | --- | --- | --- |
| **Item1** | 1.000 |  |  |  |  |  |  |  |  |  |  |  |  |  |  |
| **Item2** | 0.849^**^ | 1.000 |  |  |  |  |  |  |  |  |  |  |  |  |  |
| **Item3** | 0.543^**^ | 0.549^**^ | 1.000 |  |  |  |  |  |  |  |  |  |  |  |  |
| **Item4** | 0.624^**^ | 0.610^**^ | 0.703^**^ | 1.000 |  |  |  |  |  |  |  |  |  |  |  |
| **Item5** | 0.558^**^ | 0.582^**^ | 0.656^**^ | 0.705^**^ | 1.000 |  |  |  |  |  |  |  |  |  |  |
| **Item6** | 0.505^**^ | 0.498^**^ | 0.642^**^ | 0.669^**^ | 0.670^**^ | 1.000 |  |  |  |  |  |  |  |  |  |
| **Item7** | 0.168^**^ | 0.193^**^ | 0.202^**^ | 0.176^**^ | 0.191^**^ | 0.119^**^ | 1.000 |  |  |  |  |  |  |  |  |
| **Item8** | 0.166^**^ | 0.177^**^ | 0.197^**^ | 0.186^**^ | 0.187^**^ | 0.216^**^ | 0.719^**^ | 1.000 |  |  |  |  |  |  |  |
| **Item9** | 0.346^**^ | 0.359^**^ | 0.381^**^ | 0.372^**^ | 0.401^**^ | 0.402^**^ | 0.094^**^ | 0.195^**^ | 1.000 |  |  |  |  |  |  |
| **Item10** | 0.173^**^ | 0.229^**^ | 0.171^**^ | 0.204^**^ | 0.198^**^ | 0.212^**^ | 0.594^**^ | 0.598^**^ | 0.225^**^ | 1.000 |  |  |  |  |  |
| **Item11** | 0.394^**^ | 0.444^**^ | 0.481^**^ | 0.407^**^ | 0.454^**^ | 0.426^**^ | 0.189^**^ | 0.168^**^ | 0.531^**^ | 0.246^**^ | 1.000 |  |  |  |  |
| **Item12** | 0.387^**^ | 0.406^**^ | 0.425^**^ | 0.421^**^ | 0.427^**^ | 0.435^**^ | 0.162^**^ | 0.182^**^ | 0.509^**^ | 0.248^**^ | 0.669^**^ | 1.000 |  |  |  |
| **Item13** | 0.185^**^ | 0.214^**^ | 0.206^**^ | 0.217^**^ | 0.198^**^ | 0.178^**^ | 0.593^**^ | 0.584^**^ | 0.117^**^ | 0.649^**^ | 0.160^**^ | 0.106^**^ | 1.000 |  |  |
| **Item14** | 0.127^**^ | 0.159^**^ | 0.144^**^ | 0.149^**^ | 0.134^**^ | 0.154^**^ | 0.555^**^ | 0.563^**^ | 0.126^**^ | 0.615^**^ | 0.194^**^ | 0.190^**^ | 0.697^**^ | 1.000 |  |
| **Item15** | 0.098^**^ | 0.115^**^ | 0.091^**^ | 0.108^**^ | 0.092^**^ | 0.136^**^ | 0.446^**^ | 0.481^**^ | 0.112^**^ | 0.519^**^ | 0.131^**^ | 0.180^**^ | 0.579^**^ | 0.688^**^ | 1.000 |
| **N** | 910 | 910 | 910 | 910 | 910 | 910 | 910 | 910 | 910 | 910 | 910 | 910 | 910 | 910 | 910 |
| **Mean** | 1.83 | 1.80 | 1.51 | 1.46 | 1.49 | 1.66 | 3.07 | 3.29 | 2.23 | 3.07 | 2.09 | 2.31 | 3.15 | 3.57 | 3.81 |
| **SE** | 0.05 | 0.05 | 0.04 | 0.04 | 0.04 | 0.05 | 0.07 | 0.07 | 0.06 | 0.07 | 0.05 | 0.06 | 0.07 | 0.07 | 0.07 |
| **SD** | 1.47 | 1.49 | 1.21 | 1.16 | 1.16 | 1.40 | 1.98 | 2.08 | 1.74 | 2.08 | 1.57 | 1.82 | 2.07 | 2.04 | 2.05 |
| **Var** | 2.16 | 2.22 | 1.46 | 1.35 | 1.35 | 1.96 | 3.92 | 4.33 | 3.03 | 4.33 | 2.46 | 3.31 | 4.28 | 4.16 | 4.20 |
| **Skew** | 1.88 | 2.02 | 2.90 | 3.00 | 2.88 | 2.39 | 0.70 | 0.53 | 1.26 | 0.64 | 1.41 | 1.27 | 0.67 | 0.33 | 0.14 |
| **Kurt** | 2.80 | 3.24 | 8.56 | 9.17 | 8.43 | 5.06 | -0.67 | -1.06 | 0.41 | -0.91 | 1.11 | 0.38 | -0.86 | -1.10 | -1.20 |
| **Range** | 6.00 | 6.00 | 6.00 | 6.00 | 6.00 | 6.00 | 6.00 | 6.00 | 6.00 | 6.00 | 6.00 | 6.00 | 6.00 | 6.00 | 6.00 |

^**^p<0.05
